# Supplementary material for: Safety Evaluation of Weissella cibaria JW15 by Phenotypic and Genotypic Property Analysis
Source: Microorganisms. 2021 Nov 27;9(12):2450. doi: 10.3390/microorganisms9122450 (PMC8704421; doi:10.3390/microorganisms9122450)
Supplement: Supplementary file 1 [file microorganisms-09-02450-s001.zip › microorganisms-1457333-SI.pdf]

Tabel S1. Data of historical positive/negative controls

Historical negative control values of revertant colonies

Strain	S9 mix	N	Mean	±	S.D.	Range	
						Lower	Upper
TA100	-	70	85.7	±	9.5	60	111
	+	70	95.9	±	10.3	65	126
TA1535	-	69	11.6	±	2.1	5	18
	+	69	11.0	±	1.6	6	16
WP2*uvrA*	-	10	29.1	±	5.1	6	53
	+	10	31.6	±	2.6	20	44
TA98	-	70	18.8	±	2.9	10	28
	+	70	30.2	±	4.9	23	37
TA1537	-	69	8.4	±	1.0	5	11
	+	69	16.1	±	2.8	9	23

Historical positive control values of revertant colonies

Strain	S9 mix	Positive control	Dose (μg/plate)	N	Mean	±	S.D.	Range	
								Lower	Upper
TA100	-	SA	1.5	70	662.1	±	59.9	550	774
	+	2-AA	2.0	67	808.1	±	127.6	602	1,014
TA1535	-	SA	1.5	70	522.1	±	52.8	413	631
	+	2-AA	3.0	69	153.2	±	21.8	116	190
WP2*uvrA*	-	4-NQO	0.3	10	730.5	±	207.6	198	1,263
	+	2-AA	10.0	10	901.9	±	237.3	411	1,393
TA98	-	2-NF	5.0	70	622.9	±	92.6	506	740
	+	2-AA	1.0	67	392.0	±	52.4	280	504
TA1537	-	9-AA	80.0	70	519.1	±	92.4	372	666
	+	2-AA	3.0	66	204.1	±	31.1	131	277

Negative control: Water for injection, Dimethyl sulfoxide, Acetone, Tetrahydrofuran, *etc*.

SA: Sodium azide

2-AA: 2-Aminoanthracene

4-NQO: 4-Nitroquinoline N-oxide

2-NF: 2-Nitrofluorene

9-AA: 9-Aminoacridine

N: The total number of bacterial reverse mutation test

S.D.: Standard Deviation

The above historical control values were obtained from the data pooled from Sep. 29, 2016 to Nov. 14, 2019.

The above historical control values were obtained from the data pooled from Jan. 16, 2020 to Feb. 16, 2020 (WP2*uvrA*).

The range was calculated by the control limit of X derived from $\bar{\bar{X}}$-$\bar{X}$-$\bar{R}s$ value.
